# Single-Cell and Bulk RNA-Seq Profiling Identifies Cell Adhesion-Related Gene Signatures in Cerebral Ischemia–Reperfusion Injury

**DOI:** 10.1523/ENEURO.0209-26.2026

**Published:** 2026-10-09

**Authors:** Chengxing Qian, Luxi Qian, Zehao Fang, Jie Chen, Chuanxi Peng

**Affiliations:** ^1^Department of Neurosurgery, Tongling People's Hospital, Tongling, Anhui 244099, China; ^2^Tongling Academy of Medical Sciences, Tongling, Anhui 244099, China; ^3^Medical Imaging, Bengbu Medical University, Bengbu, Anhui 233000, China; ^4^Clinical Medicine, Youjiang Medical University for Nationalities, Baise, Guangxi 533000, China

**Keywords:** cerebral ischemia–reperfusion injury, cell adhesion-related gene, immune infiltration, single-cell RNA-seq, subtype identification

## Abstract

Cerebral ischemia–reperfusion injury (CIRI) involves complex secondary damage mechanisms, including oxidative stress, inflammation, and blood–brain barrier disruption, in which cell adhesion-related genes (CARGs) play a central role. Therefore, systematically elucidating the expression and regulation of CARGs is crucial for identifying new therapeutic targets to mitigate inflammatory infiltration and preserve barrier integrity. Both single-cell and bulk transcriptomic data were obtained from the Gene Expression Omnibus database. The single-cell data were processed using Seurat, and CARG activity was comprehensively assessed using five scoring algorithms, including UCell and AUCell. A combined machine learning framework incorporating Boruta algorithm, LASSO regression, and random forest was employed to screen for reliable diagnostic biomarkers. Furthermore, immune infiltration profiles were evaluated using CIBERSORT, regulatory networks were constructed, and molecular subtypes were identified through consensus clustering. Single-cell analysis revealed high CARG activity in endothelial cells, smooth muscle cells, and astrocytes. Machine learning identified four robust diagnostic biomarkers (Itgb1, Fn1, Pdlim5, Kdr) with excellent predictive accuracy (AUC, 1) across multiple datasets. These biomarkers strongly correlated with immune cell infiltration, particularly macrophage polarization and mast cell activation, and successfully stratified CIRI samples into two distinct molecular subtypes. By integrating single-cell and transcriptomic data, this study systematically reveals the critical role of CARGs in CIRI. It identifies four core diagnostic genes that are closely associated with immune microenvironment remodeling and neuronal damage, thereby providing new molecular markers and a theoretical foundation for clinical diagnosis and treatment.

## Significance Statement

Cerebral ischemia–reperfusion injury (CIRI) triggers persistent neuroinflammation and blood–brain barrier breakdown, yet reliable molecular biomarkers for early diagnosis and subtype stratification remain limited. Combining single-cell and bulk RNA-seq datasets, this work systematically characterizes cell adhesion-related gene (CARG) signatures in CIRI. We uncovered elevated CARG activity in endothelial cells, astrocytes, and smooth muscle cells and identified four core diagnostic genes (*Itgb1, Fn1, Pdlim5, Kdr*) with outstanding predictive performance. These genes are tightly linked to immune cell polarization and microenvironment remodeling, enabling classification of CIRI into distinct molecular subtypes. Our findings advance the understanding of cell adhesion-mediated secondary brain injury and offer promising candidate markers for future translational research and targeted therapeutic development in ischemic stroke.

## Introduction

Cerebral ischemia–reperfusion injury (CIRI) is a critical and complex pathophysiological phenomenon in the treatment of cerebrovascular diseases (Qin et al., 2025). When brain tissue becomes ischemic due to thrombosis, embolism, or systemic hypotension, timely restoration of blood flow is essential to salvage dying neurons. However, this process may trigger a series of secondary damage, leading to exacerbated brain tissue injury (Sun et al., 2018; Alsadder and Hamadah, 2025). The essence lies in the activation and amplification of accumulated potential damage during ischemia through multiple interrelated mechanisms upon reperfusion. These mechanisms mainly include a burst of reactive oxygen species production at the moment of CIRI, triggering intense oxidative stress that attacks lipids, proteins, and DNA (Medvedeva et al., 2025). CIRI can cause intracellular calcium overload, impair mitochondrial function, and activate various degrading enzymes (Li et al., 2025). It also leads to a strong inflammatory response, including the recruitment, adhesion, and infiltration of leukocytes (especially neutrophils), as well as the release of inflammatory cytokines and chemokines, resulting in microcirculatory disturbances and secondary tissue destruction (Jurcau and Simion, 2021). In addition, there is impairment of the integrity of the blood–brain barrier (BBB) and activation of cell death pathways such as apoptosis and programmed necrosis (Yang et al., 2023). This injury is not limited to the ischemic core area but can also affect the surrounding penumbra, narrowing the therapeutic time window and severely impacting neurological recovery (He et al., 2025). Therefore, a deep understanding of the cellular and molecular mechanisms of CIRI is of paramount importance for addressing CIRI.

Cell adhesion molecules, as a class of key transmembrane glycoproteins widely distributed on cell surfaces that mediate interactions between cells or between cells and the extracellular matrix, serve not only as the fundamental physical basis for maintaining the integrity of multicellular tissue structures but also as central hubs regulating transmembrane signal transduction, migration, proliferation, and immune responses (Wang et al., 2020; Jo et al., 2024). In the complex pathological progression of CIRI, the dramatic remodeling of the cell adhesion-related gene (CARG) expression profile constitutes a core pathological mechanism that initiates and amplifies the inflammatory cascade, leading to the disruption of BBB integrity (Yilmaz and Granger, 2008). When blood flow is restored to the ischemic brain tissue, reactive oxygen species and inflammatory cytokines rapidly stimulate cerebral microvascular endothelial cells, resulting in a significant upregulation in the transcription and expression levels of proinflammatory adhesion-related genes, including the selectin family and the immunoglobulin superfamily (Wu et al., 2020). These overexpressed adhesion molecules specifically recognize and bind to circulating neutrophils and monocytes, mediating their rolling, firm adhesion on the endothelial surface, and ultimately transmigration into the brain parenchyma (Wu et al., 2024). This process contributes to microcirculatory obstruction and the release of cytotoxic substances that exacerbate neuronal damage (Yuan et al., 2025). Concurrently, genes responsible for maintaining the tightness of the BBB, such as those encoding tight junction proteins and adherens junction proteins, exhibit significant transcriptional suppression or reduced protein levels under ischemic–hypoxic conditions and due to degradation by matrix metalloproteinases (Lochhead et al., 2020). This leads to the disruption of interendothelial connections and increased barrier permeability (Zhang et al., 2021). Therefore, systematically elucidating the differential expression patterns and regulatory networks of CARGs during CIRI is of substantial clinical significance for identifying novel therapeutic targets capable of inhibiting inflammatory cell infiltration and preserving BBB integrity.

Therefore, this study aims to integrate single-cell sequencing data with multicenter transcriptomic data to systematically investigate the expression patterns and underlying regulatory mechanisms of CARGs in CIRI. We employed multiple machine learning (ML) algorithms to screen key feature genes, constructed and validated clinically applicable diagnostic models, and further elucidated the intrinsic associations between these genes and immune microenvironment remodeling as well as synaptic functional impairment. This research is expected to provide novel insights into the molecular pathological mechanisms of CIRI and lay a theoretical foundation for precise clinical diagnosis, molecular subtyping, and the identification of potential therapeutic targets.

## Materials and Methods

### Ethical statement

Not applicable. This study does not include any human or animal testers.

### Data download

Download the transcriptomic data of CIRI [GSE137482 (Androvic et al., 2020)] from the GEO database (https://www.ncbi.nlm.nih.gov/geo/) as the training cohort. There are 24 samples in total, including 12 CIRI samples and 12 control samples. Additionally, download the transcriptomic data of CIRI [GSE61616 (Wang et al., 2015), GSE97537, and GSE163614 (Yi et al., 2021)] as the validation datasets. GSE61616 comprises 10 samples, including five CIRI cases and five control cases; GSE975373 comprises 12 samples, including seven CIRI cases and five control cases; GSE163614 comprises 6 samples, including three CIRI cases and three control cases. Download single-cell transcriptomic data of CIRI [GSE227651 (Zeng et al., 2023)] from the GEO database, including four samples in total: one Sham control sample and three transient middle cerebral artery occlusion samples (CIRI group). These three samples correspond to single-cell transcriptional profiles from mice on Day 1, Day 3, and Day 7 after reperfusion, respectively. Furthermore, 1,542 CARGs were obtained from the MSigDB database (https://www.gsea-msigdb.org/gsea/msigdb/; Lv et al., 2025).

### scRNA data processing

Single-cell transcriptomic data underwent quality control using the Seurat (version 4.3.0.1) software. Cell filtering criteria included removing cells with fewer than 200 or more than 3,000 genes, removing genes expressed in fewer than three cells, and excluding cells where the proportion of mitochondrial genes exceeded 10%. Low-quality cells failing to meet these criteria were discarded. Multiple samples were integrated using the merge function in the R software. To eliminate batch effects caused by sampling and experimental procedures, batch correction was performed at the sample level using the Harmony software, ensuring subsequent analyses focused more on genuine biological differences. Subsequently, 2,000 highly variable genes were selected and used as the basis for dimensionality reduction and clustering analysis.

### Dimensionality reduction, clustering, annotation, and visualization of single-cell data

The single-cell data were processed using the “Seurat” package, starting with data normalization and quality control. The top 30 principal components were extracted via RunPCA, and clustering was constructed using FindNeighbors and FindClusters, with clustree employed to compare clustering effects under different resolutions. Subsequently, differentially expressed genes (DEGs) for each cell subpopulation were identified using FindAllMarkers (min.pct = 0.25, logfc.threshold = 0.25), and annotation was performed by combining known marker genes. Finally, dimensionality reduction visualization was achieved via RunTSNE, and the expression of key genes in *t*-distributed stochastic neighbor embedding (*t*-SNE) plots and bubble plots was displayed using FeaturePlot and DotPlot.

### CARG activity score at single-cell level

To evaluate CARG activity at the single-cell level, multiple scoring methods were applied to each individual cell. UCell: A rank-based method was used to calculate CARG activity scores, enhancing the robustness of the scoring. AddModuleScore: The Seurat package was employed to compute a module score for the CARG, which integrates the expression information of the gene set into a single score, reflecting the activity of the gene set within the cell. ssGSEA: Enrichment analysis was performed on the single-cell expression profiles to obtain an enrichment score for CARG in each cell. AUCell: This method assesses CARG activity by calculating the activity of genes within each cell. Singscore: This is a rank-based scoring method that evaluates the CARG gene set for each single cell to further assess its activity level. Subsequently, the scores obtained from these methods were normalized and summed to derive a comprehensive CARG activity score. Thereby, 200 candidate genes were screened.

### Analysis of DEGs

Differential analysis was performed on the CIRI group and the control group in the training cohort using the “limma” package, with genes meeting *p*.adj < 0.01 and |log(FC)| > 1 defined as DEGs. Subsequently, the intersection of DEGs, candidate genes, and CARGs was taken to obtain differentially expressed CARGs (DECARGs). Gene Ontology (GO) and Kyoto Encyclopedia of Genes and Genomes (KEGG) enrichment analyses were conducted on the resulting intersection genes using the “clusterProfiler” package. This was followed by calculating the correlations among the intersection genes and constructing a protein–protein interaction (PPI) network using the STRING database (https://string-db.org/).

### GSVA enrichment analysis

To assess the impact of DECARGs on CIRI, we performed single-sample gene set enrichment analysis (ssGSEA) using Gene Set Variation Analysis (GSVA) on the training cohort GSE137482 and the validation datasets GSE61616, GSE97537, and GSE163614. This was done to evaluate the differences in CARG activity between the CIRI group and the Sham group.

### Screening of diagnostic biomarkers via ML

To systematically identify robust diagnostic biomarkers, this study adopted a multimodel sequential integration approach for feature screening. First, the Boruta algorithm was used for initial screening at the genome-wide level to identify candidate features with significant contributions. Subsequently, a least absolute shrinkage and selection operator (LASSO) logistic regression model was constructed to further narrow down the feature set by determining the optimal λ value through 10-fold cross-validation. Finally, a random forest (RF) model was employed to assess variable importance and select the final features. Ultimately, genes consistently identified by multiple algorithms were retained as potential diagnostic biomarkers.

### Validation of diagnostic signature genes

Receiver Operating Characteristic (ROC) curves were generated for the identified signature genes to evaluate diagnostic accuracy using the training cohort and validate them in independent validation datasets. The area under the curve (AUC) values quantified biomarker performance, and differences in the expression levels of the signature genes between the CIRI and Sham groups were examined in both the training and independent datasets.

### Immune infiltration analysis

The “IOBR” package's CIBERSORT algorithm was used to examine the immune infiltration levels in the CIRI group and the Sham group, and the differences in immune infiltration levels between the two groups were statistically analyzed. Using the “GSVA” package, ssGSEA analysis was performed to assess the differences in immune cell ssGSEA scores between the CIRI group and the Sham group, thereby investigating the disparities in immune-related functions and immune cell infiltration between the two groups.

### Single-gene enrichment analysis

Single-gene enrichment analysis is a bioinformatics method based on gene expression profile data used to analyze the correlation and regulatory mechanisms between a single gene and specific biological processes or disease states. The file “m5.go.v2025.1.Mm.symbols.gmt” was downloaded from MsigDb (https://www.gsea-msigdb.org/gsea/index.jsp), and the “GSEA” package was used to perform enrichment analysis on the signature genes.

### Construction of TF–mRNA and miRNA–mRNA regulatory networks

Upstream transcriptional regulators of the signature genes were systematically investigated using the ENCODE database via NetworkAnalyst (https://www.networkanalyst.ca/). miRNA-target gene interactions were obtained from miRTarBase (https://mirtarbase.cuhk.edu.cn/) to identify miRNAs regulating the signature genes, constructing an mRNA–miRNA regulatory network, which was visualized in Cytoscape (v3.10). Additionally, the Drug Signature Database (DSigDB, http://dsigdb.tanlab.org/DSigDBv1.0/) was used to assess potential protein–drug interactions. Specifically, target genes were uploaded to the Enrichr suite of the gene set enrichment analysis tool (https://maayanlab.cloud/modEnrichr/). Utilizing the DSigDB database, potential candidate drugs likely targeting the genes of interest were predicted.

### Identification of diagnostic gene subtypes

The CIRI samples from the combined datasets GSE137482, GSE61616, GSE97537, and GSE163614 were subjected to cluster analysis. Batch effects between datasets were corrected using the “ComBat” function from the “sva” package. The consensus clustering algorithm in the “ConsensusClusterPlus” package was then used to cluster the CIRI samples based on the obtained model signature genes, and a principal component analysis (PCA) plot was generated. Concurrently, box plots were drawn to visualize the expression of the signature genes across the different subtypes. Immune infiltration between subtypes was explored using ssGSEA. The CIBERSORT algorithm was further employed to calculate the immune infiltration levels among the subtypes. Finally, differential expression analysis was performed on the identified subtypes, and GO and KEGG pathway enrichment analyses were conducted on the resulting DEGs.

### Statistical analysis

All analyses were performed using the R language (version 4.4.1) and its associated packages. The Wilcoxon test was used to determine statistical differences between two groups. Data were primarily visualized using the “ggplot2” package. The Pearson’s method was employed to assess the correlation between two variables. Statistical significance was defined as a *p* < 0.05, with *p* values summarized as follows: ****p < 0.0001; ***0.0001 < *p* < 0.001; **0.001 < *p* < 0.01; *0.01 < *p* < 0.05; ns, *p* > 0.05.

## Results

### Evaluation of CARG-related characteristics in CIRI at the single-cell level

After rigorous data filtering and quality control, a total of 46,830 high-quality single cells from four samples (Sham group and different time points of the CIRI model) were ultimately included for subsequent analysis. Based on the maximum resolution of seurat_clusters, *t*-SNE dimensionality reduction clustering was performed, resulting in 20 clusters (Fig. 1*A*). The sample origin distribution showed that samples from each group were evenly mixed across the clusters, with no obvious batch effects observed (Fig. 1*B*). The distribution of cells from the CIRI group and the Sham group in the *t*-SNE space exhibited overall overlap but local density differences (Fig. 1*C*). Based on the expression patterns of canonical markers, we annotated these 20 subpopulations as 11 major cell types (Fig. 1*D*). It is noteworthy that in this cortical scRNA-seq dataset, endothelial cells and smooth muscle cells exhibited a relatively high representation compared with neuronal populations. While this distribution may reflect the specific cell recovery characteristics of the dissociation protocol, it provides a high-resolution window into the vascular components, which are the primary sites of cell adhesion and BBB functions. Each cell type specifically expressed its marker genes at high levels, including endothelial cells (*Cldn5, Ly6c1*), oligodendrocytes (*Opalin, Mog*), smooth muscle cells (*Acta2, Des*), choroid plexus cells (*Prlr, Clic6*), pericytes (*Atp13a5, Abcc9*), microglial cells (*Hexb, C1qa*), neurons (*Rtn1, Sox4*), astrocytes (*Gjb6, F3*), ependymal cells (*Calml4, Clu*), OPCs (*Olig2, Tnr*), and T cells (*Cd8a, Klrd1*). Compared with the Sham group, the CIRI group exhibited significant cellular composition remodeling at the early stage of injury (1d), characterized mainly by a relative decrease in the proportion of endothelial cells and alterations in the proportion of oligodendrocyte components (Fig. 1*F*). To accurately assess the activity levels of CARG in different cell types, a multialgorithm comprehensive evaluation strategy was employed. Five independent scoring methods (including UCell, AddModuleScore, ssGSEA, AUCell, and Singscore) were used to score the single-cell data (Fig. 1*G*). Although the absolute score ranges differed among the algorithms, their trends across cell types showed high consistency. To visually present these results, the composite scores were mapped back to the *t*-SNE space (Fig. 1*H*), and cells were further divided into High_CARG and Low_CARG groups based on their scores (Fig. 1*I*). Combining the results from the violin plots and feature plots, we found that endothelial cells, smooth muscle cells, and astrocytes exhibited significantly higher CARG activity. These cell types may be key effector cells in the pathophysiological processes mediated by CARG.

**Figure 1.**
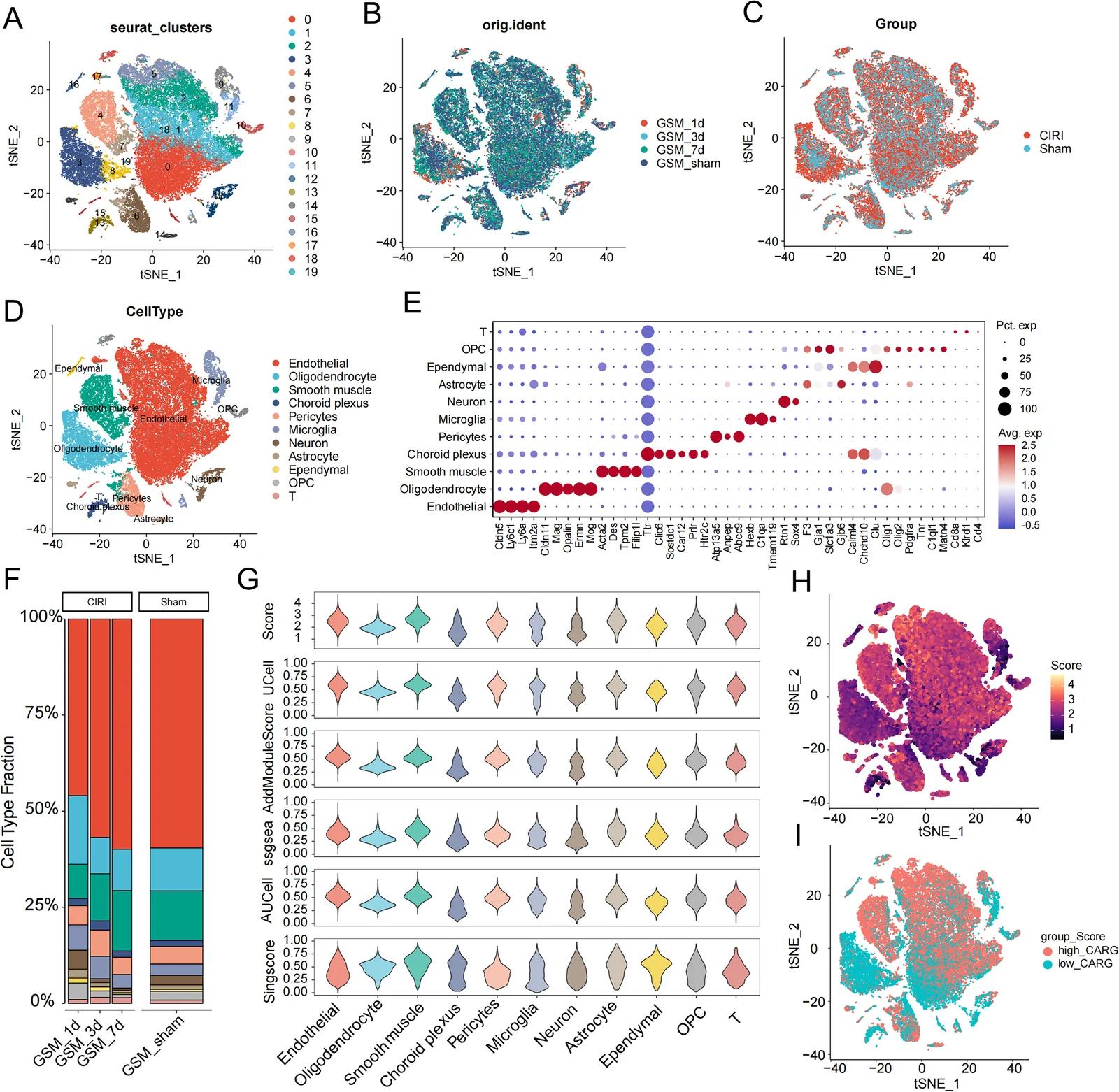
Single-cell characterization of CARG-related features in CIRI. ***A***, *t*-SNE dimensionality reduction clustering map of single-cell transcriptome data. ***B***, *t*-SNE distribution map from different samples. ***C***, *t*-SNE distribution map of the CIRI group and the Sham group. ***D***, *t*-SNE distribution map of 11 cell types. ***E***, Bubble plot showing the expression of canonical marker genes in 11 different cell types. ***F***, Relative proportion of different cell types in the CIRI group and the Sham group across different samples. ***G***, CARG activity scores for different cell types as shown by the UCell, AddModuleScore, ssGSEA, AUCell, and Singscore methods. ***H***, *t*-SNE distribution map of the total CARG activity score. ***I***, *t*-SNE distribution map after dividing the total CARG activity score into high and low groups based on the median.

### Screening for diagnostic biomarkers

To investigate the molecular regulatory network mediated by CARGs at the single-cell transcriptome level, we adopted a multidimensional gene screening strategy. First, based on the single-cell data, we calculated the correlation between gene expression and the CARGs score, and the top 200 genes with the highest positive correlation with CARGs activity were selected as the candidate set based on correlation coefficient ranking (Fig. 2*A*). Meanwhile, differential expression analysis was performed between the CIRI and Sham groups in the training cohort. The volcano plot revealed a total of 2,149 genes with significant changes, including 2,033 upregulated genes and 116 downregulated genes (Fig. 2*B*; Extended Data Fig. 2-1). To identify core molecules that are both disease-specific and closely related to CARG function, we intersected the CARG-related candidate genes, DEGs, and CARGs (Fig. 2*C*), ultimately identifying 37 DECARGs. This integrative strategy was specifically designed to mitigate potential cell-type biases inherent in single-cell datasets. By cross-referencing single-cell activity with bulk transcriptomic changes, which represent the total mRNA landscape of the cerebral cortex tissue, we ensured that the identified signatures are not merely artifacts of cell-type overrepresentation but are robustly dysregulated at the whole-tissue level. Expression pattern analysis showed that these 37 DECARGs were all significantly highly expressed in the CIRI group (Fig. 2*D*). To reveal the biological functions of these genes, GO and KEGG enrichment analyses were performed (Fig. 2*E*; Extended Data Figs. 2-2, 2-3). The results indicated that these genes were mainly enriched in pathways closely related to endothelial function and inflammatory responses, such as positive regulation of vasculature development, angiogenesis, and leukocyte transendothelial migration. Further interaction network analysis revealed tight synergistic relationships among these 37 genes. The correlation heatmap showed that they exhibited highly significant positive correlations at the transcriptional level (Fig. 2*F*). The PPI network constructed based on the STRING database further confirmed that they form a close interaction network at the protein level, with *Fn1*, *Itgb1*, *Pecam1*, etc. occupying core hub positions (Fig. 2*G*). Finally, to evaluate the efficacy of these 37 DECARGs as an overall signature in disease diagnosis, the GSVA algorithm was used to calculate the enrichment scores for each sample. In the training cohort, the GSVA score was significantly higher in the CIRI group than in the Sham group (Fig. 2*H*). More importantly, this signature was successfully validated in three independent external datasets (GSE61616, GSE97537, GSE163614), with significantly elevated scores in the CIRI groups (Fig. 2*I*). These results confirm that the gene signature composed of DECARGs possesses high robustness and clinical generalizability.

10.1523/ENEURO.0209-26.2026.f2-1 Figure 2-1 Differentially expressed genes between the CIRI group and the Sham group in the training set. Download Figure 2-1, XLS file.

10.1523/ENEURO.0209-26.2026.f2-2 Figure 2-2 GO enrichment of the 37 DE-CARGs. Download Figure 2-2, XLS file.

10.1523/ENEURO.0209-26.2026.f2-3 Figure 2-3 KEGG enrichment of the 37 DE-CARGs. Download Figure 2-3, XLS file.

**Figure 2.**
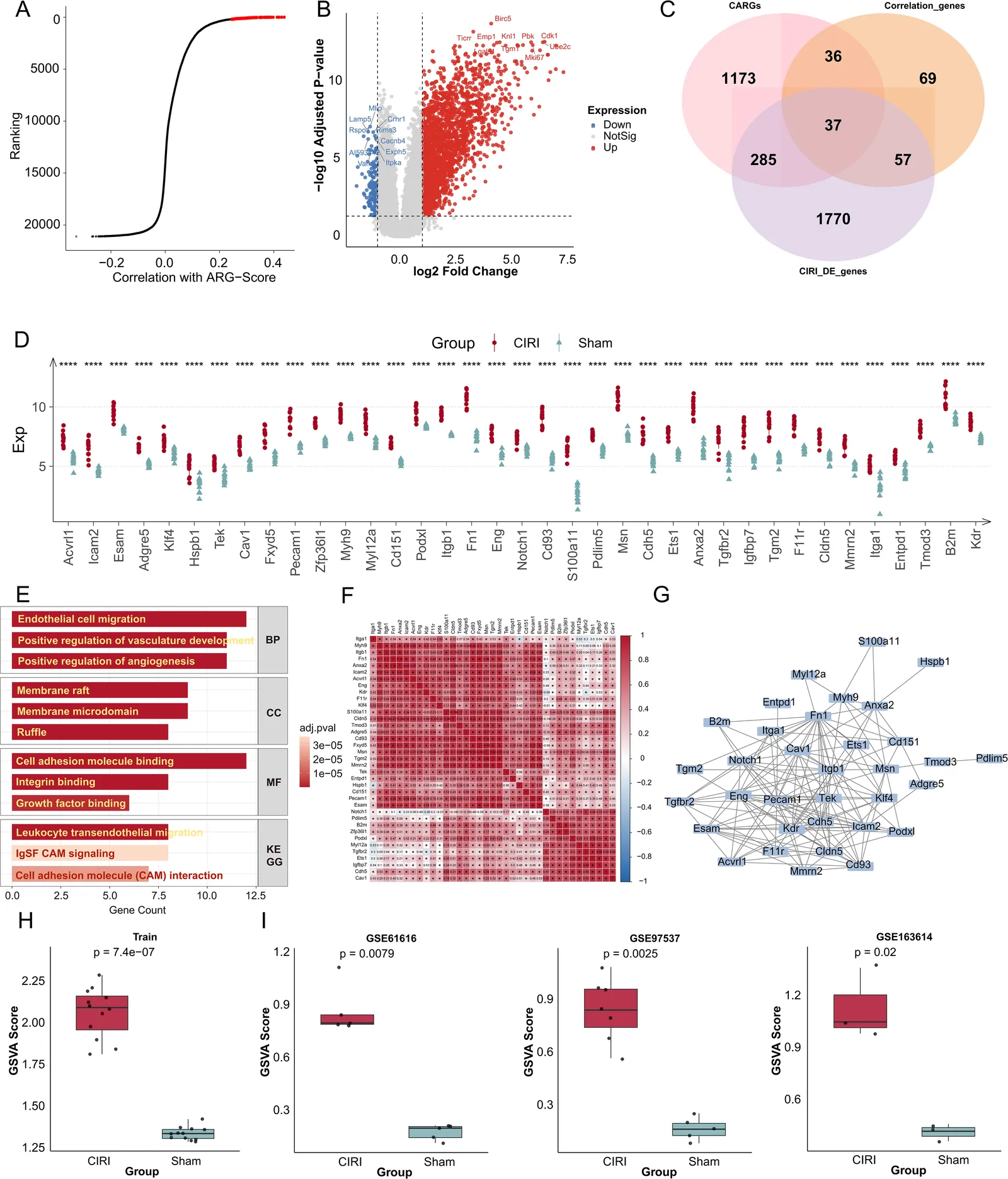
Identification of DECARGs. ***A***, Correlation analysis between single-cell gene expression and CARGs activity score. ***B***, Volcano plot of differential expression in the training cohort. ***C***, Intersection analysis of candidate genes, DEGs, and CARGs. ***D***, Expression differences of the 37 DECARGs between the CIRI group and the Sham group. ***E***, GO and KEGG enrichment analysis of the 37 DECARGs. ***F***, Correlation heatmap of the 37 DECARGs. ***G***, PPI network among the 37 DECARGs. ***H***, GSVA box plot comparing the CIRI group and the Sham group in the training cohort. ***I***, GSVA box plots comparing the CIRI group and the Sham group in the validation datasets GSE61616, GSE97537, and GSE163614. See also Extended Data Figures 2-1–2-3.

### Screening for diagnostic biomarkers using ML

To further screen for key variables from the aforementioned 37 DECARGs, variables present in both the training and validation datasets were first selected, followed by the application of three ML algorithms—Boruta, RF, and LASSO regression. Among these, Boruta evaluated feature importance using *Z*-scores, defining variables with importance significantly higher than the maximum shadow feature as “Confirmed” variables, thereby effectively removing irrelevant noise (Fig. 3*A*). RF screened key variables based on feature importance scores, and genes with a Mean Decrease Gini value >0.5 were selected as key variables (Fig. 3*B*). LASSO identified genes with nonzero coefficients under the optimal regularization parameter λ (Fig. 3*C*,*D*). Finally, four feature genes consistently selected by all three methods were identified as robust diagnostic biomarkers for subsequent modeling and validation (Fig. 3*E*).

**Figure 3.**
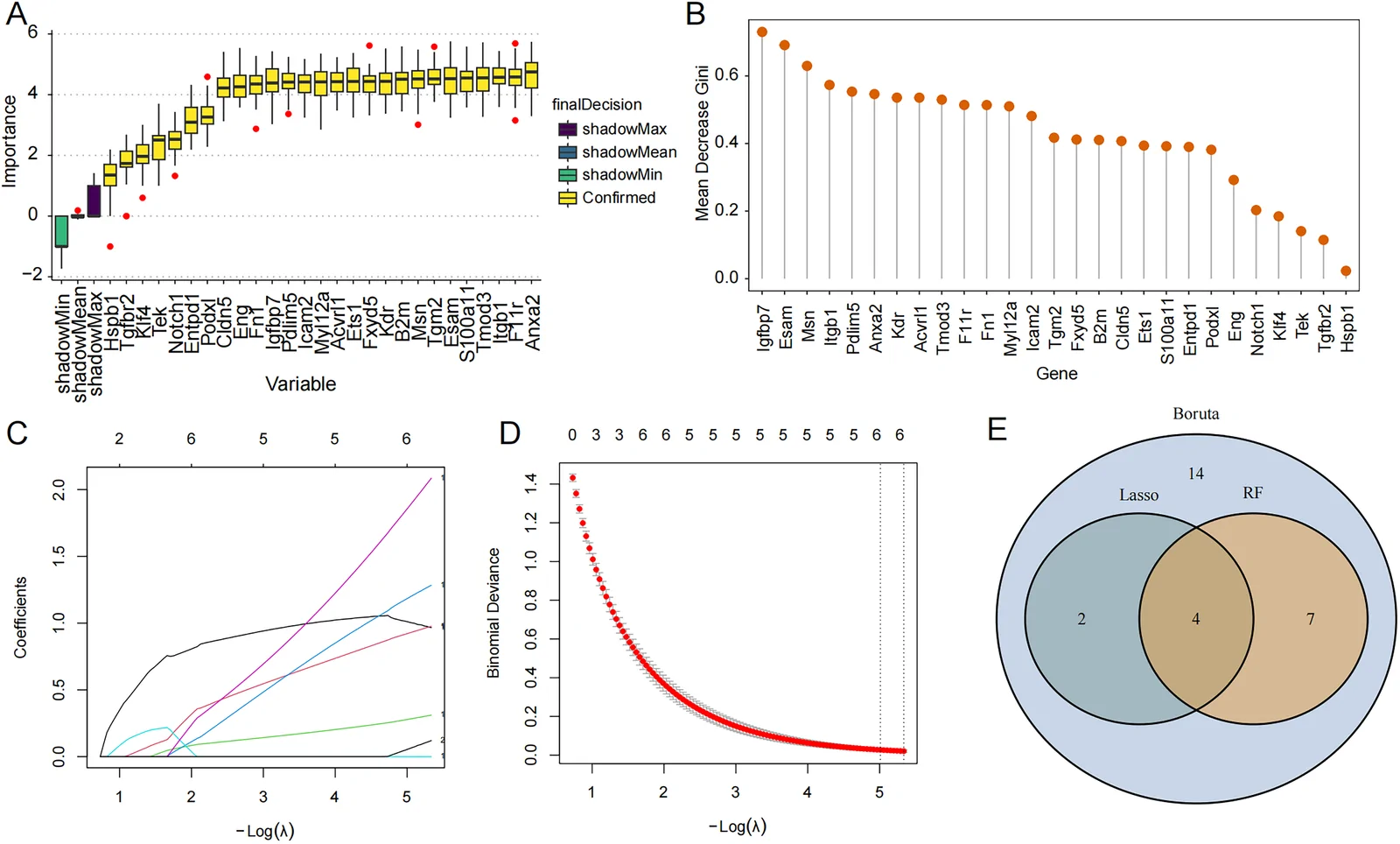
Identification of diagnostic biomarkers via ML. ***A***, Box plot of variable importance analysis using the Boruta algorithm. ***B***, RF variable importance ranking based on mean decrease in accuracy and mean decrease in Gini index. ***C***, LASSO coefficient profile from LASSO logistic analysis. ***D***, Coefficient distribution plot generated for the log(λ) sequence in the LASSO model. ***E***, Intersection analysis of the three ML algorithms.

### Validation of diagnostic biomarkers

To further evaluate the clinical application value of the aforementioned four key signature genes (*Itgb1*, *Fn1*, *Pdlim5*, *Kdr*), a visual Nomogram diagnostic model was constructed (Fig. 4*A*). The calibration curve demonstrated that the predicted probability curve of the model closely aligned with the ideal curve, indicating excellent agreement between the predicted outcomes and the actual observations (Fig. 4*B*). Additionally, the decision curve analysis (DCA) results showed that across a wide range of threshold probabilities, the net benefit of this diagnostic model was significantly higher than that of the “treat-all” or “treat-none” strategies, suggesting its promising potential for clinical application (Fig. 4*C*). ROC curve analysis revealed that in the training cohort, the multigene combined model exhibited perfect discriminative ability (AUC = 1; Fig. 4*D*). To validate the robustness of the model, it was applied to three independent external validation datasets (GSE61616, GSE97537, GSE163614). The results showed that the combined model maintained exceptionally high diagnostic performance across all validation datasets (AUC = 1 for each), outperforming or matching the predictive efficacy of any single gene. This confirms the model's strong stability and consistency across different datasets (Fig. 4*E*). Finally, in the training cohort, the expression levels of Itgb1, Fn1, Pdlim5, and Kdr were all significantly higher in the CIRI group than in the Sham group (Fig. 4*F*). This consistent upregulation was also validated in the three external validation datasets (Fig. 4*G*). To further elucidate the cellular origin of the four identified biomarkers *(Itgb1*, *Fn1*, *Pdlim5*, and *Kdr*), we mapped their expression back to the single-cell landscape. Intriguingly, these genes exhibited distinct cellular preferences within the three CARG-high cell types identified earlier. Specifically, *Itgb1* and *Pdlim5* showed peak expression levels in smooth muscle cells, while *Fn1* and *Kdr* were predominantly localized in endothelial cells (Extended Data Fig. 4-1). This cellular compartmentalization suggests that the four diagnostic markers may reflect damage and remodeling across different components of the neurovascular unit.

**Figure 4.**
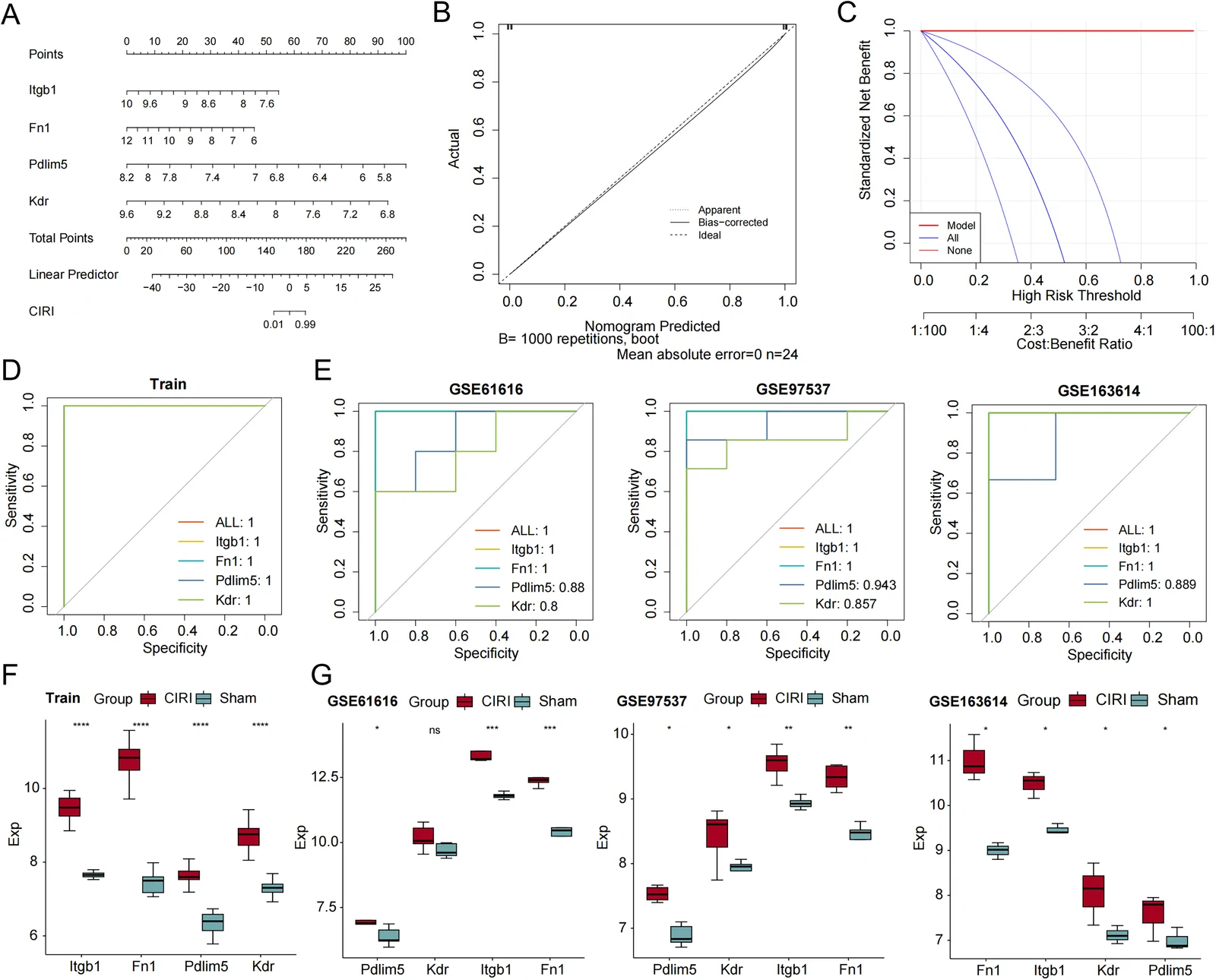
Diagnostic model development and validation. ***A***, Nomogram of the signature genes in the diagnostic model. ***B***, Calibration curve of the nomogram. ***C***, DCA curve of the diagnostic model. ***D***, ROC curves of the diagnostic model and individual signature genes in the training cohort. ***E***, ROC curves of the diagnostic model and individual signature genes in the validation datasets. ***F***, Box plots of signature gene expression in the CIRI group versus the Sham group in the training cohort. ***G***, Box plots of signature gene expression in the CIRI group versus the Sham group in the validation datasets. See also Extended Data Figure 4-1.

10.1523/ENEURO.0209-26.2026.f4-1 Figure 4-1 Cell-type specific expression profiles of the four diagnostic hub genes. Download Figure 4-1, DOCX file.

### Immune infiltration analysis

Given the central role of immune-inflammatory responses in the pathological progression of CIRI, we systematically evaluated the infiltration abundance of immune cells in the CIRI group versus the Sham group using the CIBERSORT algorithm (Fig. 5*A*). The results revealed a significant remodeling of the immune microenvironment in CIRI tissues compared with the Sham group. The CIRI group exhibited significantly increased infiltration of Macrophages M1, Macrophages M2, and Mast cells activated. Conversely, the proportions of Mast cells resting and T cells CD4 memory resting were markedly reduced after injury (Fig. 5*B*). The ssGSEA algorithm quantified the enrichment scores of immune-related functions (Fig. 5*C*). The CIRI group showed significantly higher scores than the Sham group in pathways such as Inflammation-promoting, APC co-stimulation, Macrophages, and Type I IFN Response (Fig. 5*D*). This further confirms the presence of robust innate immune activation and antigen-presenting activity in the local microenvironment of CIRI. To investigate whether the four core characteristic genes identified are involved in the remodeling of the aforementioned immune microenvironment, the correlation between gene expression levels and immune cell abundance was calculated. These four genes exhibited strikingly similar immune-related patterns: they all showed significant positive correlations with Macrophages M1, Macrophages M2, and Mast cells activated while displaying significant negative correlations with Mast cells resting and T cells CD4 memory resting (Fig. 5*E*–*H*). This result strongly suggests that these characteristic genes may play a key regulatory role in the immune-inflammatory injury mechanism of CIRI by mediating the recruitment and activation of macrophages and mast cells.

**Figure 5.**
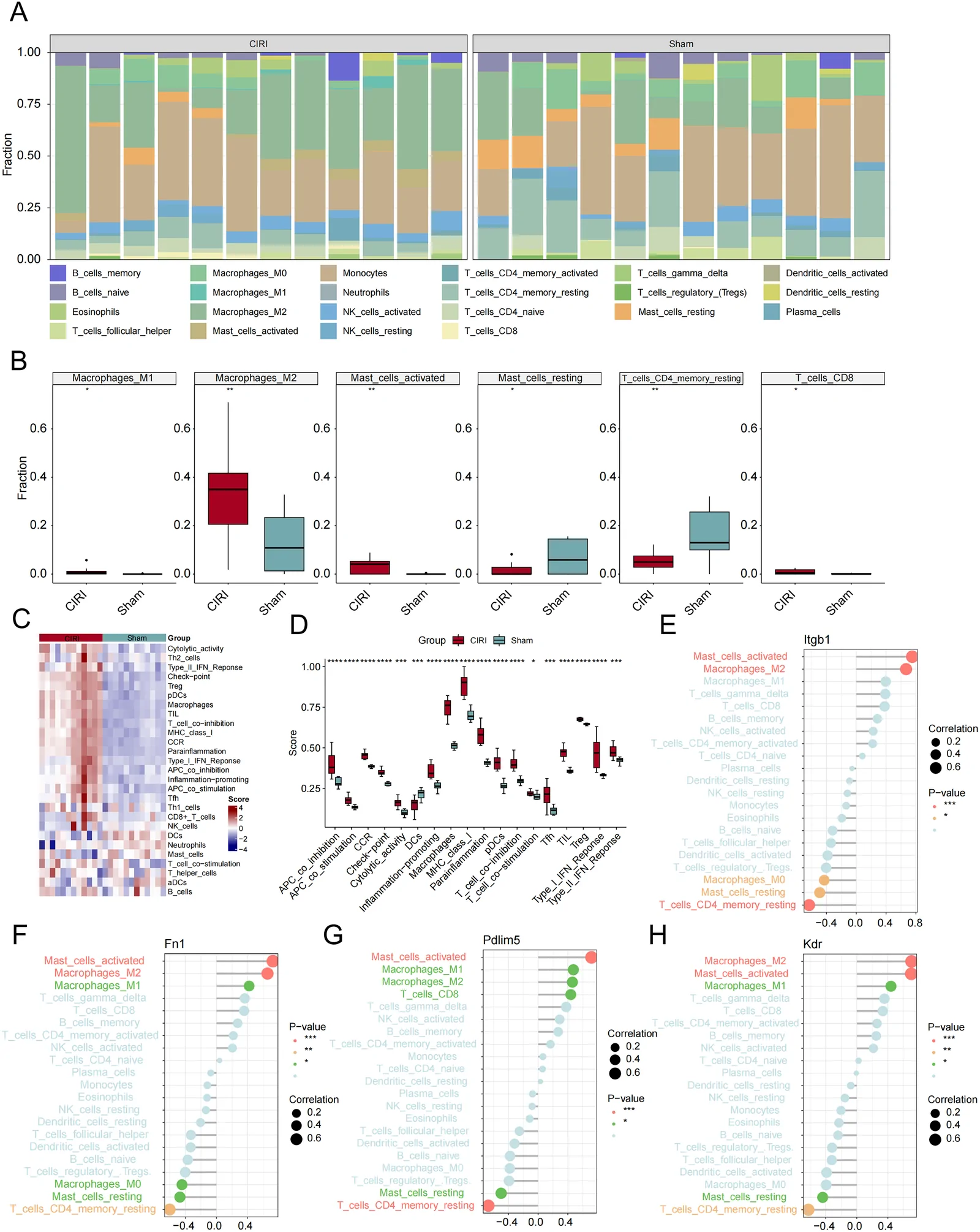
Immune microenvironment characterization. ***A***, Distribution of immune cell types in the CIRI group and the control group. ***B***, Difference diagram of immune cell type distribution between the CIRI group and the control group. ***C***, Heatmap of immune infiltration levels between the CIRI group and the control group. ***D***, Differences in immune infiltration levels between the CIRI group and the control group. Correlation between *ITGB1* (***E***), *FN1* (***F***), *PDLIM5* (***G***), and *KDR* (***H***) gene expression and immune cell infiltration levels.

### Single-gene enrichment analysis

Single-gene GSEA was performed to analyze the GO functional enrichment profiles of key diagnostic biomarkers. Within the activated pathways, all four genes showed significant enrichment in immune- and inflammation-related biological processes, such as positive regulation of interleukin-1 production, positive regulation of inflammatory response, leukocyte chemotaxis, and adaptive immune response. This indicates that high expression of these genes is closely associated with intense local inflammatory storms and immune cell infiltration after CIRI. Among the suppressed pathways, these genes were significantly negatively correlated with processes related to neurotransmission and synaptic structure. The inhibited pathways mainly included excitatory synapse, postsynaptic membrane, GABAergic synapse, and dendrite membrane. These results suggest that elevated expression of these diagnostic biomarkers significantly suppresses normal synaptic transmission in the nervous system, reflecting neuronal functional impairment induced by CIRI (Fig. 6*A*–*D*).

**Figure 6.**
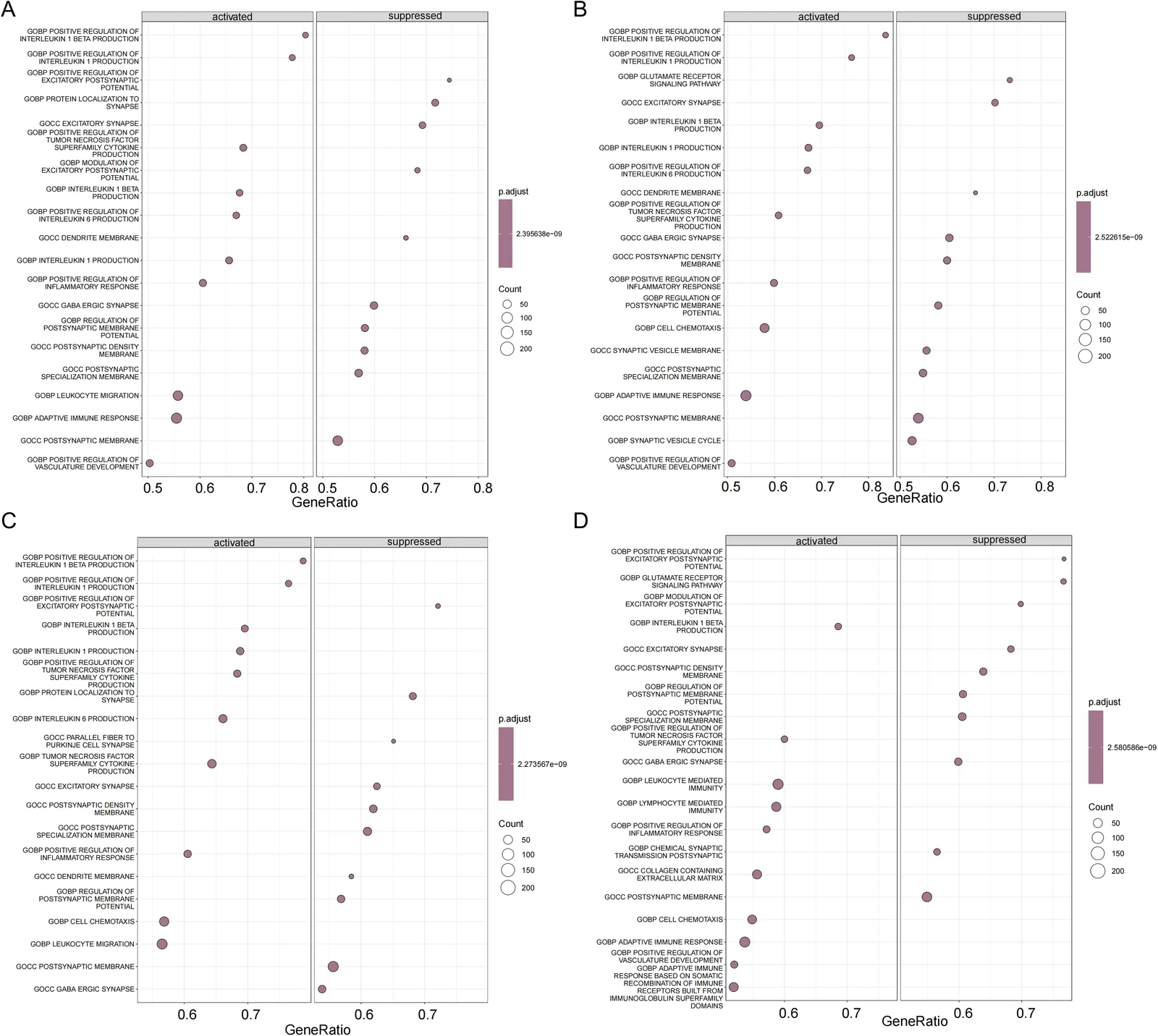
Results of single-gene enrichment analysis. GO enrichment of *Itgb1* (***A***), *Fn1* (***B***), *Pdlim5* (***C***), and *Kdr* (***D***) signature genes.

### Constructing a regulatory network associated with signature genes

Based on the NetworkAnalyst platform and the ENCODE database, we identified and constructed a TF–-mRNA regulatory network (Fig. 7*A*). The analysis revealed that *Pdlim5* and *Itgb1* occupy central positions in the regulatory network and are targeted by multiple classical transcription factors. Specifically, the transcription factors *SIN3A* and *CHD1* both show regulatory connections with *Pdlim5* and *Itgb1*. These shared transcriptional regulatory nodes suggest that these signature genes may be coregulated through specific transcription factor complexes, thereby jointly participating in the pathological process of CIRI. Using the miRTarBase database, miRNAs targeting these signature genes were predicted, and an mRNA-miRNA interaction network was constructed (Fig. 7*B*). *Fn1* exhibited the broadest miRNA-binding potential, with predicted targeting molecules including mmu-miR-1a-3p, mmu-miR-206-3p, and mmu-miR-29b-1-5p, among others. Itgb1 was mainly regulated by molecules such as mmu-miR-26a-5p and mmu-miR-223-3p, while *Pdlim5* was identified as a target gene of miRNAs including mmu-miR-2136. Furthermore, the top five candidate drugs predicted by DSigDB are listed in Table 1 (Extended Data Fig. 7-1).

**Figure 7.**
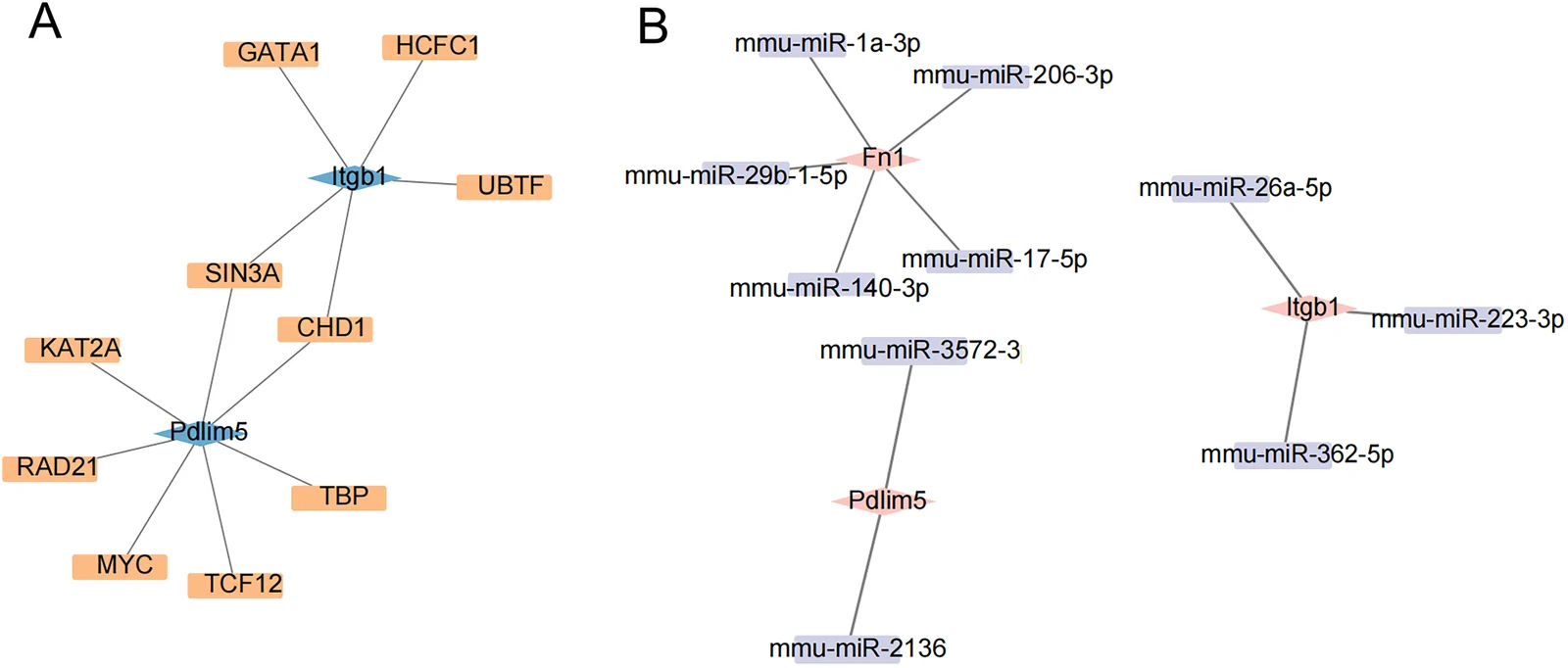
Construction of the signature gene regulatory network. ***A***, TF network of the model signature genes. Orange nodes represent transcription factors, blue nodes represent signature genes, and connecting lines indicate regulatory relationships. ***B***, miRNA network of the model signature genes. Red nodes represent signature genes, purple nodes represent miRNAs, and connecting lines indicate regulatory relationships. See also Extended Data Figure 7-1.

10.1523/ENEURO.0209-26.2026.f7-1 Figure 7-1 Candidate drugs predicted by the DSigDB for the signature genes. Download Figure 7-1, XLS file.

**Table 1.** Drug prediction for model signature genes

Term	*p* value	Adjusted *p* value	Odds ratio	Combined score	Genes
LAMININ BOSS	3.73755930398401e-06	0.000895600535307995	306.216494845361	3,826.81134505392	*ITGB1;FN1;KDR*
Acid red 87 BOSS	3.7949175224915e-06	0.000895600535307995	304.630769230769	3,802.35492127542	*ITGB1;FN1;KDR*
HEMATOXYLIN BOSS	7.74035663702675e-06	0.00121781611089221	238.887096774194	2,811.47724268086	*ITGB1;FN1;KDR*
*N*-Acetyl-l-cysteine CTD 00005305	1.52996717285682e-05	0.00178010634958289	189.269230769231	2,098.55651044522	*ITGB1;KDR;PDLIM5*
trolox CTD 00000557	1.88570587879544e-05	0.00178010634958289	587.117647058824	6,387.03168146862	*FN1;KDR*

### CIRI subtype analysis based on diagnostic signature genes

To expand the sample size and validate the generalizability of the findings, we integrated four datasets: GSE137482, GSE61616, GSE97537, and GSE163614. Following batch-effect correction, PCA demonstrated thorough mixing of samples across datasets, indicating effective removal of nonbiological variations (Fig. 8*A*,*B*). Using the four core signature genes identified, we performed unsupervised consensus clustering on CIRI samples via the ConsensusClusterPlus algorithm and determined *k* = 2 as the optimal cluster number, thereby stratifying CIRI samples into two subtypes with distinct molecular profiles (Fig. 8*C*–*E*). Among them, Cluster 1 comprised 11 samples and Cluster 2 comprised 16 samples. PCA further confirmed a clear separation between these two subtypes at the transcriptomic level (Fig. 8*F*). Comparison of signature gene expression levels revealed that all four signature genes (*Fn1*, *Itgb1*, *Kdr*, *Pdlim5*) were significantly upregulated in Cluster 1 (Fig. 8*G*). Differential expression analysis between Cluster 1 and Cluster 2 identified 227 upregulated and 548 downregulated genes (Cluster 1 vs Cluster 2; Fig. 8*H*). Enrichment analysis showed that upregulated genes were significantly associated with pathways such as leukocyte migration, collagen-containing extracellular matrix, and cytokine–receptor interactions (Fig. 8*I*). Downregulated genes were mainly enriched in biological processes related to normal tissue metabolism, including organic anion transport, hormone activity, and fat digestion and absorption (Fig. 8*J*). ssGSEA analysis indicated that scores for the majority of immune cell infiltration and inflammation-related pathways (e.g., Type I IFN Response, Inflammation-promoting) were significantly higher in Cluster 1 than in Cluster 2 (Fig. 8*K*). Analysis of specific cell fractions with CIBERSORT revealed that Cluster 1 exhibited higher proportions of Mast cells activated and Macrophages M2 infiltration, whereas Cluster 2 showed significantly higher proportions of Macrophages M1 and T cells CD4 memory resting (Fig. 8*L*).

**Figure 8.**
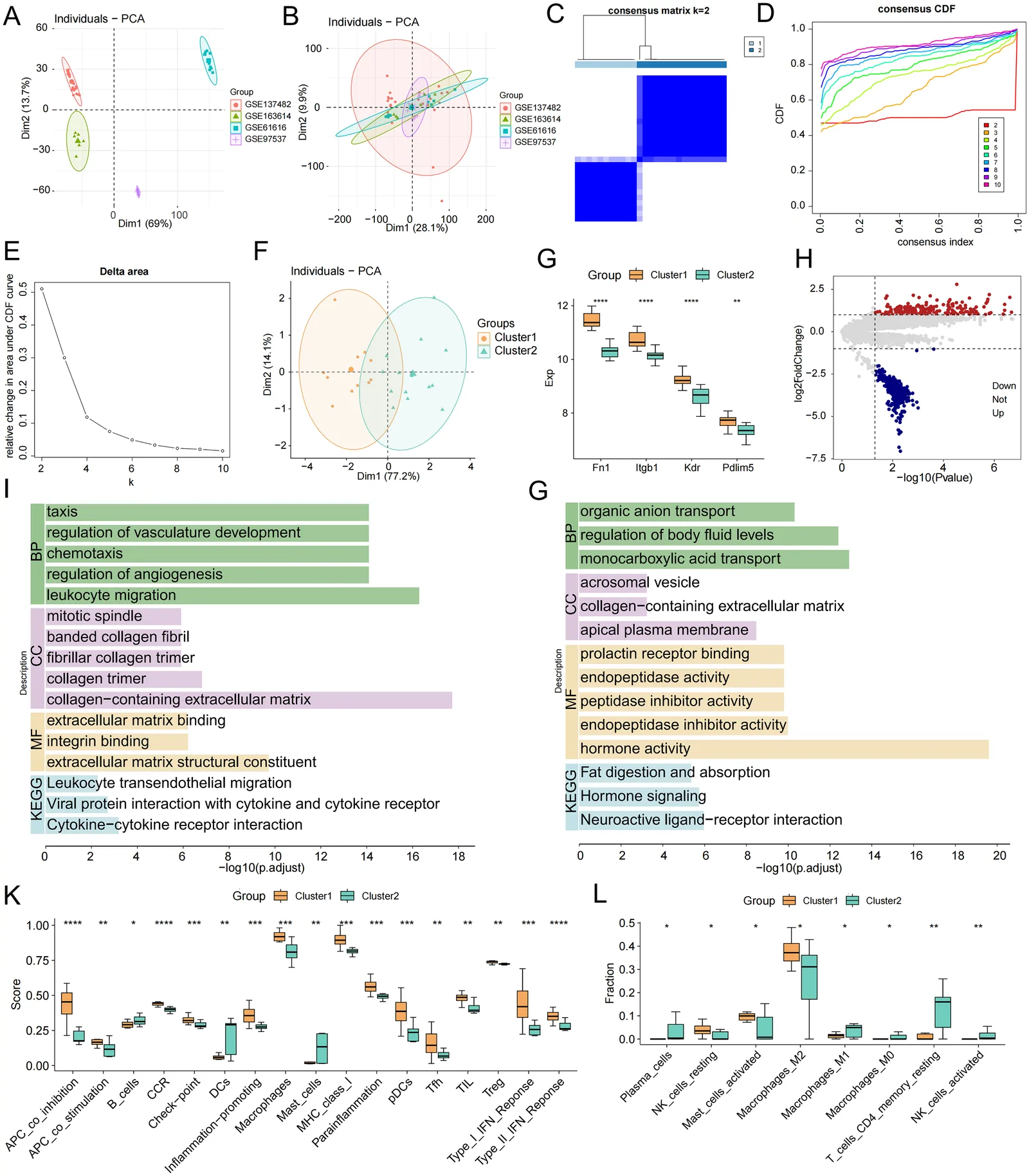
Subtype identification and correlation analysis. ***A***, PCA plot before batch-effect removal. ***B***, PCA plot after batch-effect removal. ***C***, Consensus clustering analysis by ConsensusClusterPlus. ***D***, Cumulative distribution function (CDF) plot for cluster stability. ***E***, Relative change in area under the CDF. ***F***, PCA plot of CIRI subtypes. ***G***, Box plot of diagnostic gene expression across subtypes. ***H***, Volcano plot of differential expression between subtypes. ***I***, GO/KEGG enrichment analysis of upregulated genes. ***J***, GO/KEGG enrichment analysis of downregulated genes. ***K***, Box plot comparing immune cell/functional activity across subtypes. ***L***, Box plot comparing immune cell infiltration across subtypes.

## Discussion

This study systematically elucidates the expression landscape and core regulatory networks of CARGs in the pathological progression of CIRI by integrating single-cell transcriptomics data with multicenter, large-sample bulk transcriptome sequencing data. We not only revealed the specific high-activity states of CARGs in endothelial cells, smooth muscle cells, and astrocytes at single-cell resolution but also successfully identified and established a robust diagnostic signature composed of *Itgb1*, *Fn1*, *Pdlim5*, and *Kdr*. The research findings provide a deep analysis of the intrinsic link between cell adhesion molecule-mediated remodeling of the immune microenvironment and synaptic function impairment. It confirms the clinical value of this gene signature in distinguishing disease subtypes and predicting potential drug targets, thereby offering a new theoretical foundation and molecular strategy for the precise diagnosis and targeted intervention of CIRI.

Single-cell transcriptome analysis not only finely delineates the microenvironment atlas of CIRI at cellular resolution but also deeply reveals the pathogenic mechanisms of CARGs from the perspective of the overall homeostasis of the neurovascular unit. We found that CARG activity is specifically enriched in endothelial cells, smooth muscle cells, and astrocytes. While the scRNA-seq dataset utilized here showed a higher representation of vascular-related cells compared with neurons, this distribution provides an ideal opportunity to investigate cell adhesion, which is fundamentally a vascular-mediated process in CIRI. Importantly, we mitigated potential sampling bias by intersecting single-cell candidates with bulk RNA-seq data, which represents the entire cortical tissue. This integrative approach ensures that our hub genes are not artifacts of cell-type overrepresentation but are truly representative of the ischemic cortical pathology. Endothelial cells, as the structural and functional basis of the BBB, exhibit high CARG activity, which directly reflects alterations in barrier permeability (Kaya and Ahishali, 2021). After reperfusion, stress-induced cytoskeletal remodeling in endothelial cells initially widens intercellular gaps, leading to initial leakage. Subsequently, upregulation of adhesion molecules recruits leukocytes, which release proteases and free radicals, amplifying the damage. Ultimately, repair signals such as Wnt determine whether the BBB can be reconstructed (Shi et al., 2016). The active CARG in astrocytes further confirms their key role in maintaining the neuronal microenvironment, mediating neuroinflammation, and facilitating glial scar formation (Cieri and Ramos, 2025).

Through systematic screening, this study identified *Itgb1*, *Fn1*, *Pdlim5*, and *Kdr* as core hub genes. Our single-cell localization analysis revealed a distinct compartmentalization of these biomarkers: *Itgb1* and *Pdlim5* exhibited peak expression in SMCs, whereas *Fn1* and *Kdr* were predominantly enriched in ECs. This finding suggests that these diagnostic markers capture different facets of neurovascular remodeling. *ITGB1 (Integrin subunit beta 1)* is a transmembrane glycoprotein-coding gene widely expressed in neurons, oligodendrocytes, microglia, and endothelial cells in the brain. It regulates synaptic plasticity, myelination, and neuroinflammatory responses (Chang et al., 2021; Huang et al., 2025). The THY1 signaling in neurons maintains the quiescent state of astrocytes via ITGB1-mediated transcellular signaling dialogue, thereby facilitating BBB repair (Loui et al., 2026). Astrocytes and endothelial cells highly express *Fn1*, which suppresses the VEGF, HBEGF, and IGF1 pathways through integrin-FAK signaling, disrupts astrocyte-endothelial cell interactions, and increases BBB permeability (Bhattarai et al., 2025). Additionally, the expression of Pdlim5 increases after reperfusion. Its PDZ domain binds and sequesters phosphorylated YAP and TAZ in the cytoplasm, reduces TEAD transcriptional activity, decreases tight junction protein synthesis, and increases paracellular permeability of the BBB (Zhang et al., 2024). Based on the findings, this study identifies *Itgb1*, *Fn1*, and *Pdlim5* as crucial regulators in post-CIRI BBB dysfunction, operating through mechanisms of neurovascular communication, matrix signaling, and junctional protein modulation, respectively.

Our study, through an in-depth analysis of immune cell infiltration patterns, reveals significant remodeling features of the immune microenvironment following CIRI. The results demonstrate a marked increase in the infiltration of M1 macrophages, M2 macrophages, and activated mast cells in CIRI tissues, while the proportions of resting mast cells and resting memory CD4^+^ T cells are significantly reduced. This suggests an early shift from adaptive immunity toward innate immunity dominance upon reperfusion (L et al., 2016). This transition is likely associated with the release of local damage-associated molecular patterns and the recruitment of peripheral immune cells after BBB disruption (Wang et al., 2025). Postischemia–reperfusion, the DAMPs-TLR4/Dectin-1-NF-κB/STAT1 axis drives macrophages toward M1 polarization, rapidly releasing TNF-α, IL-1β, and ROS/NO, thereby triggering a proinflammatory cascade and neuronal apoptosis. This represents a core mechanism underlying acute-phase neurological damage in CIRI (Zongyun et al., 2021).

This study still has certain limitations. All findings are primarily based on public database mining and bioinformatics analysis; the expression patterns of the core diagnostic genes and the predicted drugs have yet to be validated in independent clinical tissues or animal models through wet-lab experiments such as RT-qPCR and Western blot. The sample size of the existing datasets is relatively small, which somewhat limits the generalizability and clinical translation potential of the conclusions. Moreover, the proposed TF–mRNA/miRNA–mRNA regulatory networks and potential drug targets mainly rely on algorithmic predictions. Their specific molecular mechanisms and regulatory effects on the immune microenvironment still require further elucidation through in vitro and in vivo functional experiments, such as gene knock-out or overexpression.

## Conclusion

By integrating single-cell and transcriptomic data, this study has elucidated the crucial role of CARGs in CIRI. We observed that these genes are highly active in endothelial cells, smooth muscle cells, and astrocytes, and we identified four core diagnostic genes. These genes have been confirmed to be closely associated with immune microenvironment remodeling and neurological functional impairment. The findings of this research provide new molecular markers and theoretical foundations for the clinical diagnosis and targeted therapy of CIRI.

## Data Availability

The data and materials in the current study are available from the corresponding author on reasonable request.

## Synthesis

Reviewing Editor: Selva Baltan, Oregon Health &Science University Department of Surgery Decisions are customarily a result of the Reviewing Editor and the peer reviewers coming together and discussing their recommendations until a consensus is reached. When revisions are invited, a fact-based synthesis statement explaining their decision and outlining what is needed to prepare a revision will be listed below. The following reviewer(s) agreed to reveal their identity: NONE.

Dear Authors,

The review of your manuscript has generated interest and assessed as a study that advances the field. It is also recognized for the appropriate statistical analysis of the findings. However, please respond to each of the comments I will share with you during your revision. Make sure your interpretation of the results is updated based on the requested approach. The revised version will be shared with the reviewer(s) for final decision.

Thanks,

Selva Baltan

## References

[B1] Alsadder L, Hamadah A (2025) Cardiac ischaemia-reperfusion injury: pathophysiology, therapeutic targets and future interventions. Biomedicines 13:2084. 10.3390/biomedicines13092084 PMC12467651 41007647

[B2] Androvic P, et al. (2020) Decoding the transcriptional response to ischemic stroke in young and aged mouse brain. Cell Rep 31:107777. 10.1016/j.celrep.2020.107777 32553170

[B3] Bhattarai P, et al. (2025) APOE- ε4-induced Fibronectin at the blood-brain barrier is a conserved pathological mediator of disrupted astrocyte-endothelia interaction in Alzheimer’s disease. *bioRxiv*.

[B4] Chang R, Xiao X, Fu Y, Zhang C, Zhu X, Gao Y (2021) ITGB1-DT facilitates lung adenocarcinoma progression via forming a positive feedback loop with ITGB1/Wnt/β-catenin/MYC. Front Cell Dev Biol 9:631259. 10.3389/fcell.2021.631259 PMC7982827 33763420

[B5] Cieri MB, Ramos AJ (2025) Astrocytes, reactive astrogliosis, and glial scar formation in traumatic brain injury. Neural Regen Res 20:973–989. 10.4103/NRR.NRR-D-23-02091 PMC11438322 38989932

[B6] He Y, Huang P, Li S, Lin J, Ren X, Xiong Y, Yang Y, Hong D (2025) Exploration of the therapeutic time window for thrombectomy in rat models of middle cerebral artery ischemia-reperfusion. Brain Behav 15:e70713. 10.1002/brb3.70713 PMC12321963 40760823

[B7] Huang T, Fakurazi S, Cheah PS, Ling KH (2025) Dysregulation of REST and its target genes impacts the fate of neural progenitor cells in down syndrome. Sci Rep 15:2818. 10.1038/s41598-025-87314-y PMC11754635 39843579

[B8] Jo Y, Sim HI, Yun B, Park Y, Jin HS (2024) Revisiting T-cell adhesion molecules as potential targets for cancer immunotherapy: CD226 and CD2. Exp Mol Med 56:2113–2126. 10.1038/s12276-024-01317-9 PMC11541569 39349829

[B9] Jurcau A, Simion A (2021) Neuroinflammation in cerebral ischemia and ischemia/reperfusion injuries: from pathophysiology to therapeutic strategies. Int J Mol Sci 23:14. 10.3390/ijms23010014 PMC8744548 35008440

[B10] Kaya M, Ahishali B (2021) Basic physiology of the blood-brain barrier in health and disease: a brief overview. Tissue Barriers 9:1840913. 10.1080/21688370.2020.1840913 PMC7849738 33190576

[B11] L L, X W, Z Y (2016) Ischemia-reperfusion injury in the brain: mechanisms and potential therapeutic strategies. Biochem Pharmacol (Los Angel) 5:213. 10.4172/2167-0501.1000213 PMC5991620 29888120

[B12] Li YW, Liu Y, Luo SZ, Huang XJ, Shen Y, Wang WS, Lang ZC (2025) The significance of calcium ions in cerebral ischemia-reperfusion injury: mechanisms and intervention strategies. Front Mol Biosci 12:1585758. 10.3389/fmolb.2025.1585758 PMC12104078 40421420

[B13] Lochhead JJ, Yang J, Ronaldson PT, Davis TP (2020) Structure, function, and regulation of the blood-brain barrier tight junction in central nervous system disorders. Front Physiol 11:914. 10.3389/fphys.2020.00914 PMC7424030 32848858

[B14] Loui J, Krügel U, Winkler U, Reinert A, John D, Hirrlinger J, Saalbach A (2026) Neuronal THY1 signaling maintains astrocytes in a quiescent state. Glia 74:e70083. 10.1002/glia.70083 PMC12666997 40936326

[B15] Lv DM, Yang L, Fan C, Fang LH, Cheng SF (2025) Identification of cell adhesion-related subtypes and construction of risk model to predict breast cancer prognostic and immunological properties. World J Surg Oncol 23:152. 10.1186/s12957-025-03802-5 PMC12013005 40264099

[B16] Medvedeva YV, Sharman E, Weiss JH (2025) Mechanisms of delayed ischemia/reperfusion evoked ROS generation in the hippocampal CA1 zone of adult mouse brain slices. Sci Rep 15:23439. 10.1038/s41598-025-07070-x PMC12222531 40604107

[B17] Qin X, Zhu J, Lu H, Yi M, Zhao Z, Zhang W, Cheng J (2025) Research progress of deubiquitinating enzymes in cerebral ischemia-reperfusion injury. Front Aging Neurosci 17:1588920. 10.3389/fnagi.2025.1588920 PMC12171222 40529211

[B18] Shi Y, et al. (2016) Rapid endothelial cytoskeletal reorganization enables early blood-brain barrier disruption and long-term ischaemic reperfusion brain injury. Nat Commun 7:10523. 10.1038/ncomms10523 PMC4737895 26813496

[B19] Sun MS, Jin H, Sun X, Huang S, Zhang FL, Guo ZN, Yang Y (2018) Free radical damage in ischemia-reperfusion injury: an obstacle in acute ischemic stroke after revascularization therapy. Oxid Med Cell Longev 2018:3804979. 10.1155/2018/3804979 PMC5892600 29770166

[B20] Wang H, Yiqiang Z, Cai J, Guo J (2025) Immune-mediated blood-brain barrier disruption after ischemic stroke: mechanisms and therapeutic targets. Front Immunol 16:1716041. 10.3389/fimmu.2025.1716041 PMC12727575 41451205

[B21] Wang L, Yu Y, Yang J, Zhao X, Li Z (2015) Dissecting Xuesaitong’s mechanisms on preventing stroke based on the microarray and connectivity map. Mol Biosyst 11:3033–3039. 10.1039/C5MB00379B 26305988

[B22] Wang Z, Xu Q, Zhang N, Du X, Xu G, Yan X (2020) CD146, from a melanoma cell adhesion molecule to a signaling receptor. Signal Transduct Target Ther 5:148. 10.1038/s41392-020-00259-8 PMC7421905 32782280

[B23] Wu L, Xiong X, Wu X, Ye Y, Jian Z, Zhi Z, Gu L (2020) Targeting oxidative stress and inflammation to prevent ischemia-reperfusion injury. Front Mol Neurosci 13:28. 10.3389/fnmol.2020.00028 PMC7066113 32194375

[B24] Wu X, et al. (2024) Astrocyte-derived extracellular vesicular miR-143-3p dampens autophagic degradation of endothelial adhesion molecules and promotes neutrophil transendothelial migration after acute brain injury. Adv Sci (Weinh) 11:e2305339. 10.1002/advs.202305339 PMC10837358 38044319

[B25] Yang C, Wang Y, Wu X, Gong M, Li Y (2023) Reduced levels of A20 protein prompted RIPK1-dependent apoptosis and blood-brain barrier breakdown during cerebral ischemia reperfusion injury. PLoS One 18:e0290015. 10.1371/journal.pone.0290015 PMC10424866 37578944

[B26] Yi D, Wang Q, Zhao Y, Song Y, You H, Wang J, Liu R, Shi Z, Chen X, Luo Q (2021) Alteration of N (6) -methyladenosine mRNA methylation in a rat model of cerebral ischemia-reperfusion injury. Front Neurosci 15:605654. 10.3389/fnins.2021.605654 PMC8009187 33796004

[B27] Yilmaz G, Granger DN (2008) Cell adhesion molecules and ischemic stroke. Neurol Res 30:783–793. 10.1179/174313208X341085 PMC2748428 18826804

[B28] Yuan B, Zhang X, Liu L, Chai Y, Zhang J, Chen X (2025) Neutrophils-astrocyte interactions in central nervous system inflammation. Cell Death Dis 16:643. 10.1038/s41419-025-07945-x PMC12379155 40854886

[B29] Zeng F, Cao J, Hong Z, Liu Y, Hao J, Qin Z, Zou X, Tao T (2023) Single-cell analyses reveal the dynamic functions of Itgb2(+) microglia subclusters at different stages of cerebral ischemia-reperfusion injury in transient middle cerebral occlusion mice model. Front Immunol 14:1114663. 10.3389/fimmu.2023.1114663 PMC10098327 37063847

[B30] Zhang Q, Zheng M, Betancourt CE, Liu L, Sitikov A, Sladojevic N, Zhao Q, Zhang JH, Liao JK, Wu R (2021) Increase in blood-brain barrier (BBB) permeability is regulated by MMP3 via the ERK signaling pathway. Oxid Med Cell Longev 2021:6655122. 10.1155/2021/6655122 PMC8026308 33859779

[B31] Zhang XL, et al. (2024) Glial growth factor 2 treatment alleviates ischemia and reperfusion-damaged integrity of the blood-brain barrier through decreasing Mfsd2a/caveolin-1-mediated transcellular and Pdlim5/YAP/TAZ-mediated paracellular permeability. Acta Pharmacol Sin 45:2241–2252. 10.1038/s41401-024-01323-7 PMC11489722 38902501

[B32] Zongyun C, Bixia L, Fadian D, Xiaoping H, Hongbin C, Yu D, Qicai L, Xiaoyi Y, Kai Z (2021) The role of dectin-1-mediated M1 macrophage polarization in cerebral ischemia-reperfusion injury. Emerg Med Int 2021:6697271. 10.1155/2021/6697271 PMC8163548 34094602

