# Supplementary material for: Single-Cell and Bulk RNA-Seq Profiling Identifies Cell Adhesion-Related Gene Signatures in Cerebral Ischemia–Reperfusion Injury
Source: eNeuro. 2026 Oct 9;13(10):ENEURO.0209-26.2026. doi: 10.1523/ENEURO.0209-26.2026 (PMC13657665; doi:10.1523/ENEURO.0209-26.2026)
Supplement: Supplementary file 1 [file eneuro-13-ENEURO.0209-26.2026-s005.docx]

**Extended Data Figure 4-1**

**Extended Data Figure 4-1. Cell-type specific expression profiles of the four diagnostic hub genes.**
